# An Urban Case of Chromoblastomycosis in the United States

**DOI:** 10.7759/cureus.13136

**Published:** 2021-02-04

**Authors:** Victor A Canela, Carley Legan

**Affiliations:** 1 Internal Medicine, Methodist Health System, Dallas, USA

**Keywords:** chromoblastomycosis, itraconazole, fonsecaea, tropical, cutaneous

## Abstract

Rash is a common chief complaint in the emergency room. Infection and pathogen colonization of the skin are among the most common causes of rash. Workers throughout the world are occupationally exposed to fungal species, increasing the likelihood of infection. Chromoblastomycosis is a common tropical infection found in workers exposed to the fungal species *Fonsacaea *and *Cladophialophora*. Although rarely seen in the United States, some cases of chromoblastomycosis have been reported. Presentations in immunocompetent patients primarily involve dermal infections. Long-term treatment is required to avoid complications related to untreated infection. Our case presents an elderly immunocompetent patient who emigrated from an endemic region in Asia and presented with chromoblastomycosis. This case highlights the importance of internists recognizing a locally rare but treatable condition.

## Introduction

Illnesses caused by environmental and occupational exposures are a diagnostic and preventive challenge for primary care providers and the general internist. Making a connection between an exposure and an illness can be challenging given that presentations often vary in severity and chronicity. In certain parts of the United States and the developing world, exposure to contaminants such as infectious agents or toxins can have initial cutaneous presentations [1,2]. Chromoblastomycosis is a common tropical infection found in humans exposed to the fungal species *Fonsacaea* and *Cladophialophora*; it is rarely seen in the United States. In this case report, we present the case of an elderly immunocompetent patient who emigrated from an endemic region in Asia and presented with chromoblastomycosis. This article was previously presented as a meeting abstract at the Hospital Medicine 2020 Virtual Competition.

## Case presentation

A 91-year-old healthy Taiwanese female presented with a progressing skin lesion on her right forearm. The patient, originally from Taiwan, immigrated to the United States in 1995. Initially, she had mild skin breakdown that matured to erythema, swelling, and rubor. The patient noted some purple spots on her lower extremities, especially around her right knee. The patient denied any swollen lymph nodes, fever, chills, night sweats, or weight loss. The patient stated her cheeks developed redness, but attributed this to high blood pressure. According to the patient’s family, the exact chronicity of the rash was unknown as she usually wore long sleeves and pants. The patient’s home medications included hydrochlorothiazide and meloxicam as well as hydrocodone/acetaminophen as needed.

**Figure 1:**
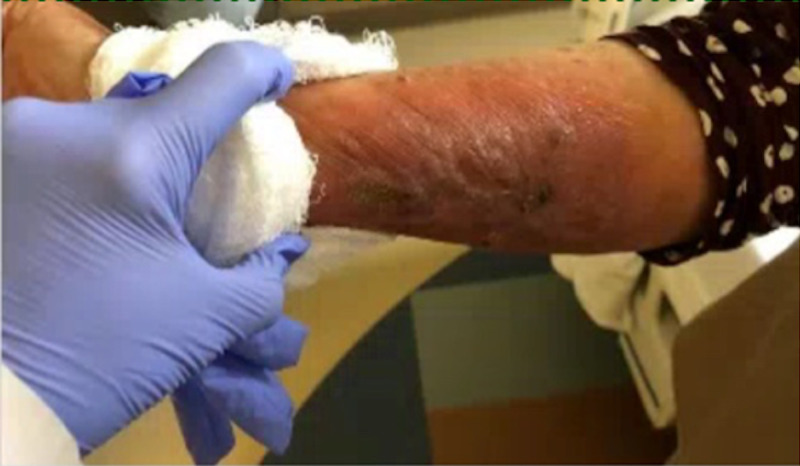
Chromoblastomycosis of the right forearm.

**Figure 2:**
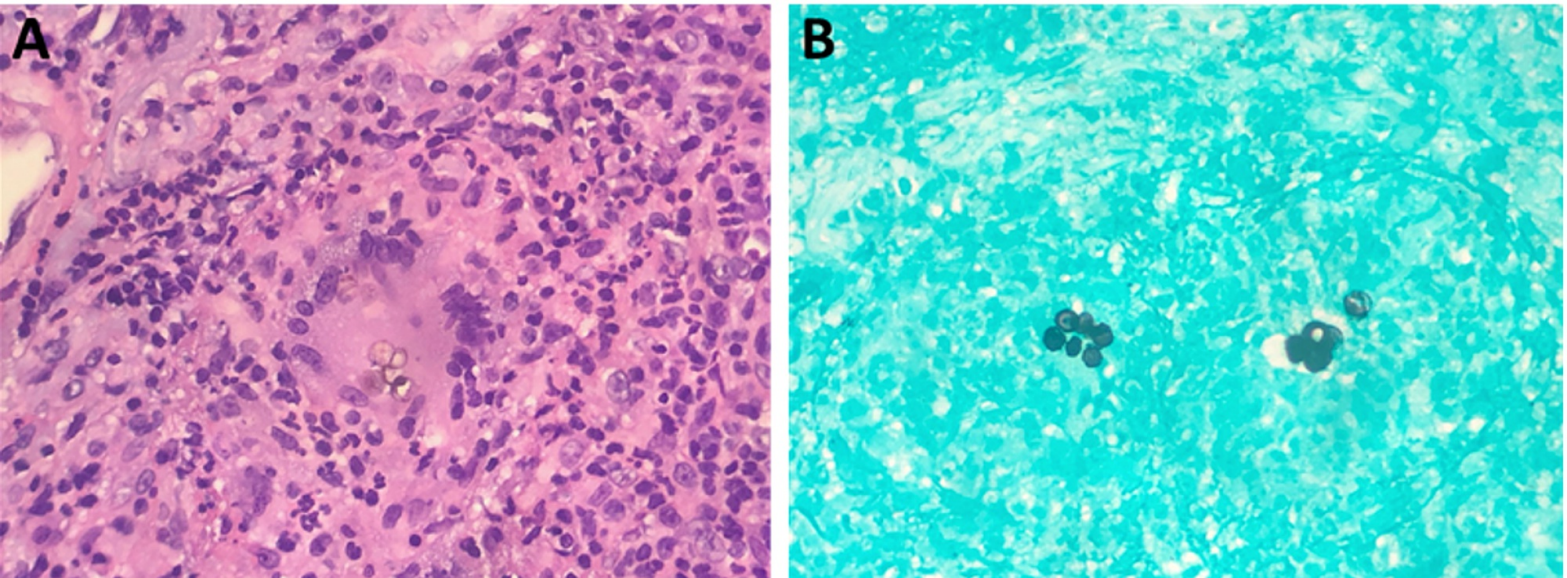
Biopsy of the forearm lesion showed sclerotic bodies using periodic acid-Schiff stain (A) and Grocott’s methenamine silver stain (B).

Upon examination, the patient was afebrile and had normal vital signs. There was a large fungated/crusted lesion present on the right forearm with a scabbed area and surrounding erythema without fluctuance or purulence (Figure 1). The lesion was mildly warm to the touch. On the patient’s lower extremities there were non-blanching purple macules, the largest located around her right knee and right thigh. No clavicular or inguinal swollen lymph nodes were noted. The initial complete blood count workup was unrevealing: prothrombin time and partial thromboplastin time were within normal range; serum and urine protein electrophoresis were normal; and antineutrophil cytoplasmic antibodies and cryoglobulins were negative. A skin biopsy of the thigh showed non-specific purpura. The nodular erythematous area on her right arm was biopsied. Findings were consistent with *Fonsecaea *species (Figure 2). The infectious disease team was consulted and the patient was started on itraconazole twice a day. Approximately one week later, she was discharged with close clinic follow-up and inflammatory markers were routinely monitored. She had a persistent rash with moderate improvement when evaluated two months later.

## Discussion

Chromoblastomycosis cases in the United States are rare. Available data suggest that the incidence of this condition in the United States is 1:8,625,000 patients [3]. Chromoblastomycosis is a chronic granulomatous disease caused by transcutaneous inoculation of fungal spores including *Fonsecaea *and *Cladophialophora* species found in tropical and subtropical regions of developing countries in Asia, Africa, and Latin America [3]. The most common agents are *F. pedrosoi* and *C. carrionii*. Chromoblastomycosis is primarily considered an occupational disease that affects immunocompetent individuals [3]. The strongest risk factor is cutaneous trauma in workers exposed to contaminated material. The infection has a varied presentation and identification of fungal elements is needed to confirm diagnosis. It is important to exclude and distinguish causes with similar presentation including other infectious agents, cutaneous malignancy, or autoimmune conditions affecting involving the skin.

It is possible that the patient was inadvertently exposed and inoculated to fungal spores while gardening in Taiwan. Initial cutaneous chromoblastomycosis lesions are often misdiagnosed and late presentations are common, resulting in a more challenging treatment. Most cases report a presentation mainly affecting young male workers and farmers from developing countries due to exposure from contaminated soil or plants [4,5]. More than half of chromoblastomycosis lesions occur in the lower extremities [4]. This case highlights one of the oldest reported immunocompetent affected females from an endemic tropical area. The very localized nature of her infection in an easily covered body part led us to believe that the patient had this infection for years before her family brought her to seek medical care.

Chromoblastomycosis diagnosis is made by identification of muriform cells from clinical samples [6,7]. Treatment for chromoblastomycosis can be challenging and varies from surgical excision of localized small lesions to systemic antifungals such as itraconazole. Modalities such as laser therapy are also reserved for more severe cases. Long-term assessment with histology is needed for up to two years to confirm cure. Inadequate treatment can result in chronic soft tissue fibrosis, chronic lymphedema, secondary infection, or the development of malignancy.

## Conclusions

Chromoblastomycosis is a common tropical fungal infection affecting occupational workers. Although rarely observed in the United States, it is important for the internist to consider this diagnosis during a rash evaluation. While the treatment is challenging, successful cure will prevent potential complications.
